# The Effect of Cesium Incorporation on the Vibrational and Elastic Properties of Methylammonium Lead Chloride Perovskite Single Crystals

**DOI:** 10.3390/ma17122862

**Published:** 2024-06-12

**Authors:** Syed Bilal Junaid, Furqanul Hassan Naqvi, Jae-Hyeon Ko

**Affiliations:** School of Nano Convergence Technology, Nano Convergence Technology Center, Hallym University, Chuncheon 24252, Gangwondo, Republic of Korea; syedbilaljunaid@hallym.ac.kr (S.B.J.); furqanhassan@hallym.ac.kr (F.H.N.)

**Keywords:** lead halide perovskites, MA_x_Cs_1−x_PbCl_3_, Raman spectroscopy, Brillouin spectroscopy

## Abstract

Hybrid organic-inorganic lead halide perovskites (LHPs) have emerged as a highly significant class of materials due to their tunable and adaptable properties, which make them suitable for a wide range of applications. One of the strategies for tuning and optimizing LHP-based devices is the substitution of cations and/or anions in LHPs. The impact of Cs substitution at the A site on the structural, vibrational, and elastic properties of MA_x_Cs_1−x_PbCl_3_-mixed single crystals was investigated using X-ray diffraction (XRD) and Raman and Brillouin light scattering techniques. The XRD results confirmed the successful synthesis of impurity-free single crystals, which exhibited a phase coexistence of dominant cubic and minor orthorhombic symmetries. Raman spectroscopy was used to analyze the vibrational modes associated with the PbCl_6_ octahedra and the A-site cation movements, thereby revealing the influence of cesium incorporation on the lattice dynamics. Brillouin spectroscopy was employed to investigate the changes in elastic properties resulting from the Cs substitution. The incorporation of Cs cations induced lattice distortions within the inorganic framework, disrupting the hydrogen bonding between the MA cations and PbCl_6_ octahedra, which in turn affected the elastic constants and the sound velocities. The substitution of the MA cations with smaller Cs cations resulted in a stiffer lattice structure, with the two elastic constants increasing up to a Cs content of 30%. The current findings facilitate a fundamental understanding of mixed lead chloride perovskite materials, providing valuable insights into their structural and vibrational properties.

## 1. Introduction

Hybrid organic-inorganic lead halide perovskites (LHPs) are based on the ABX_3_ stoichiometry with CH_3_NH_3_ (methylammonium, MA) or CH(NH_2_)_2_ (formamidinium, FA) or Cs (cesium) cations at the A site, Pb at the B site, and Cl, Br, or I at the X site. The remarkable compositional flexibility of these perovskites allows for extensive control in tuning and optimizing the physical properties. Consequently, they have gained significant attention in the photovoltaic and optoelectronic fields [1]. In particular, LHPs exhibit low trap density [2], large absorption coefficients [3] and long carrier diffusion lengths [2], making them highly suitable for photodetection applications [4,5]. Furthermore, their low-cost solution processability [6] alongside excellent hole and electron conductivity [7] make them promising candidates for integration into solar cells and light-emitting diodes (LEDs). Recent studies have highlighted the exceptional performance of LHP-based solar cells, which have achieved record power conversion efficiencies (PCEs) of over 25% [8,9] and LEDs with an external quantum efficiency (EQE) of 23.4% [10]. Furthermore, Zia et al. developed perovskite solar cells (PSCs) that were annealed in a MACl vapor atmosphere, which demonstrated superior performance compared to those that were annealed in nitrogen and air atmospheres. PSCs treated with MACl vapor exhibited the highest open-circuit voltage (V_oc_) of 1.78 V with consistent short-circuit current density (J_sc_), fill factors (FFs), and PCE values of 0.87% [11]. Nevertheless, despite the exceptional attributes of the pure perovskite compounds MAPbX_3_, FAPbX_3,_ and CsPbX_3_ (X = Br, Cl or I), challenges remain in ensuring the moisture and thermal stability critical to prolonging the lifetime of perovskite photovoltaic devices [12,13].

MAPbI_3_ is a material that is widely used in photovoltaic applications. However, achieving a PCE above 20% [7,14] has proven challenging, along with issues of thermal and moisture instability [12,13,15,16]. FAPbI_3_ has a reduced bandgap compared to MAPbI_3_, which is approaching the optimum level for single junction applications [17]. This allows for the achievement of higher potential solar efficiencies. However, it exhibits structural instability at room temperature and tends to crystallize into the undesirable yellow δ-phase [18,19,20], which is susceptible to solvents and moisture [21]. In contrast, pure CsPbBr_3_ lacks the bandgap required for photovoltaic applications. CsPbI_3_ has a bandgap of 1.73 eV [19,22], crystallizes into a photoinactive yellow phase at room temperature [19], rendering it unsuitable for practical applications. Consequently, due to the thermal or structural instabilities observed in pure perovskite compounds, researchers have endeavored to address this challenge through various approaches, including encapsulation [23], polymer coatings [24], and the substitution of A-site cations [25,26] and X-site anions [27,28] with other ions. Nevertheless, the incorporation of mixed cations and halides has attracted considerable attention due to their remarkable effectiveness in overcoming these challenges.

Grätzel et al. developed a successful method to enhance the PCEs of perovskite solar cells by synthesizing MA_0.6_FA_0.4_PbI_3_ perovskite, where the MA^+^ ions in MAPbI_3_ were partially substituted by FA^+^ ions. This substitution resulted in an improvement in efficiency compared to the parent perovskites [25]. Similarly, Zhang et al. substituted Cs cations in CsPbBr_3_ with MA^+^ and achieved an EQE of ~10.4% using Cs_0.87_MA_0.13_PbBr_3_ as an active layer in LEDs [26]. Choi et al. incorporated Cs cations into the MAPbI_3_ structure, resulting in a stabilized perovskite film and a PCE of 8% [29]. Lee et al. substituted Cs cations in FAPbI_3_ to improve thermal and moisture stability and achieved a PCE of 16.5% [30]. Yi et al. employed Cs cations to entropically stabilize the black phase in FAPbI_3_ perovskite, thereby improving its structural stability [31]. McMeekin et al. partially substituted FA cations with Cs to achieve superior thermal stability for Cs_0.17_FA_0.83_Pb(I_0.83_Br_0.17_)_3_ [32]. Saliba et al. used CsI in solar cell materials to improve the structural and thermal stability of photovoltaic devices. A combination of Cs/MA/FA cations was synthesized, resulting in perovskite solar cells with a high and stabilized PCE of 21.1% [33]. Ibaceta-Jaña et al. investigated the incorporation of cesium into FAPbI_3_ and FAPbBr_3_, which resulted in a red-shift of the low-frequency vibrational modes, indicating a release of stresses of the Pb-X bonds [34]. The incorporation of the cesium cation stabilizes the cubic structure, thereby reducing lattice stress and enhancing the overall stability of the structure. Moreover, the effect of cation incorporation on hydrogen bonding plays a vital role in the structural stability of the material and its mechanical properties. Svane et al. investigated the influence of cations on the strength and dynamics of hydrogen bonding using solid-state 1H nuclear magnetic resonance (NMR) measurements and density functional theory (DFT) calculations [35]. Their findings indicated that stronger hydrogen bonding is correlated with lower degrees of cation motion, while compounds having weaker hydrogen bonding exhibited higher cation motion. Similarly, Yin et al. conducted a comprehensive analysis of the MA orientations and hydrogen bonding dynamics in MAPbBr_3_ using Raman spectroscopy, powder X-ray diffraction, and ab initio calculations [36]. Their findings revealed that the rotation of the MA cations and tilting of the PbBr_6_ octahedra were driven by hydrogen bonding interactions between the H atoms of the CH_3_/NH_3_^+^ group and Br atoms. These interactions vary with different phases, affecting structural stability and leading to transitions characterized by the Pb-Br-Pb bond angle.

Despite the extensive previous work that has been conducted on the MA_x_Cs_1−x_PbCl_3_ mixed crystal, the study of this particular material is relatively rare. The objective of this research was to enhance our comprehension of these understudied MA_x_Cs_1−x_PbCl_3_ single crystals. To achieve this, we investigated the changes in the optical and acoustic phonon modes at room temperature by substituting 10%, 20%, and 30% of Cs at the A-site cation of MAPbCl_3_, employing Raman and Brillouin spectroscopic techniques. Both Raman and Brillouin spectroscopies belong to the inelastic light scattering caused by the interaction of incident photons and the optic and acoustic phonons whose wavevectors are located near the center of the first Brillouin zone. The obtainable information is the phonon energy, phonon lifetime, and possible coupling between the phonons and other low-energy excitations. Since the phonons are near the zone center, their wavelengths are on the order of a few hundred nanometers, indicating Raman and Brillouin scattering probes long-wavelength phonons. This is in contrast to the inelastic neutron or inelastic x-ray scattering, where the phonon dispersion curves over the whole Brillouin zone can be investigated. Since phonons are sensitive to interatomic potentials and bond strengths, the effect of Cs on the vibrational and acoustic properties can be investigated in detail. This will assist us in investigating the impact of cesium incorporation on the vibrational modes of PbCl_6_ octahedra and the interaction with the organic MA cation. While Raman spectroscopy provides us with insights into the vibrational characteristics of materials, Brillouin spectroscopy can be used to secure complementary data on the elastic behavior of these materials. The combination of these techniques is expected to enhance our understanding of the interrelationship between the structural modification induced by mixing methylammonium and cesium at the A site and phonon properties. This understanding is of significant importance for the optimization of the material in order to enhance its performance in energy-related applications. This study is expected to enhance our understanding of the interrelationship between the structural modification induced by Cs and phonon properties, which hold significant implications for the optimization of the material to enhance its performance in energy-related applications.

## 2. Experimental Section

### 2.1. Precursors

Lead chloride (PbCl_2_, 99.999%), methylamine hydrochloride (MACl, ≥98%), cesium chloride (CsCl, ≥99.999%), dimethyl sulfoxide (DMSO, anhydrous ≥ 99.9%), and N-Methyl formamide (NMF, 99%) were purchased from Sigma Aldrich (St. Louis, MO, USA). All of these chemicals were used as received without any further purification.

### 2.2. Single-Crystal Synthesis

The synthesis of MA_x_Cs_1−x_PbCl_3_ single crystals with nominal compositions of x = 0.9, 0.8, and 0.7 was performed using the solvent evaporation method. Equimolar solutions of the MACl, CsCl, and PbCl_2_ were dissolved in NMF (5 mL) and DMSO (5 mL) by stirring at 40 °C. Once complete dissolution was achieved, the solution was filtered through a 0.22 μm syringe filter into a crystallization dish. The dish was then covered with aluminum foil, and a few holes were punched in the foil to allow for slow evaporation, which resulted in better crystallization. The dish was then kept undisturbed at a constant temperature of 95 °C for 2–3 days. After a period of 2–3 days, transparent crystals of MA_x_Cs_1−x_PbCl_3_ were obtained. The crystals were then cleaned with dichloromethane and dried in a vacuum oven at 60 °C overnight.

### 2.3. Characterization Techniques

The powder XRD pattern was obtained using a high-resolution XRD spectrometer (PANalytical, Malvern, UK; X’pert PRO MPD) at the Cu K radiation wavelength (λ = 1.5406 Å). The 2θ angular range was set from 10 to 60°, and the measurement was observed at room temperature (RT). The measurement was conducted on the crystalline powder obtained following the crushing of the single crystals prior to the measurement. The XRD patterns were further analyzed using PANalytical software (X’pert Highscore v1.1).

The Raman measurements were performed on the single crystal using a standard Raman spectrometer (LabRam HR800, Horiba Co., Kyoto, Japan). The single crystal was irradiated with a diode-pumped solid-state laser at a wavelength of 532 nm, spanning a frequency range from 10 to 3500 cm^−1^. The spectrometer was equipped with a low-frequency notch filter, which permitted the measurement of frequencies as low as 10 cm^−1^. All measurements were conducted using an optical microscope (BX41, Olympus, Tokyo, Japan) with a 50× magnification objective lens with backscattering geometry. The Raman spectrometer was calibrated with a silicon standard sample exhibiting a peak at 520.7 cm^−1^. The intensities of all the measured Raman spectra were corrected for the Bose–Einstein thermal factor.

A standard tandem multi-pass Fabry–Perot interferometer (TFP-2, JRS Co., Zurich, Switzerland) with a 532 nm excitation source was employed to obtain Brillouin spectra of the single crystal. Backscattering geometry (BH-2, Olympus, Tokyo, Japan) with a modified microscope was employed for the measurement. A conventional photon counter connected to a multi-channel analyzer was employed to identify the signal and average it over 1024 channels. In order to encompass both the longitudinal acoustic (LA) and the transverse acoustic (TA) modes within the Brillouin spectrum, the free spectral range was set to 30 GHz.

## 3. Results and Discussions

### 3.1. Structure of the Grown Mixed Crystals

**Figure 1:**
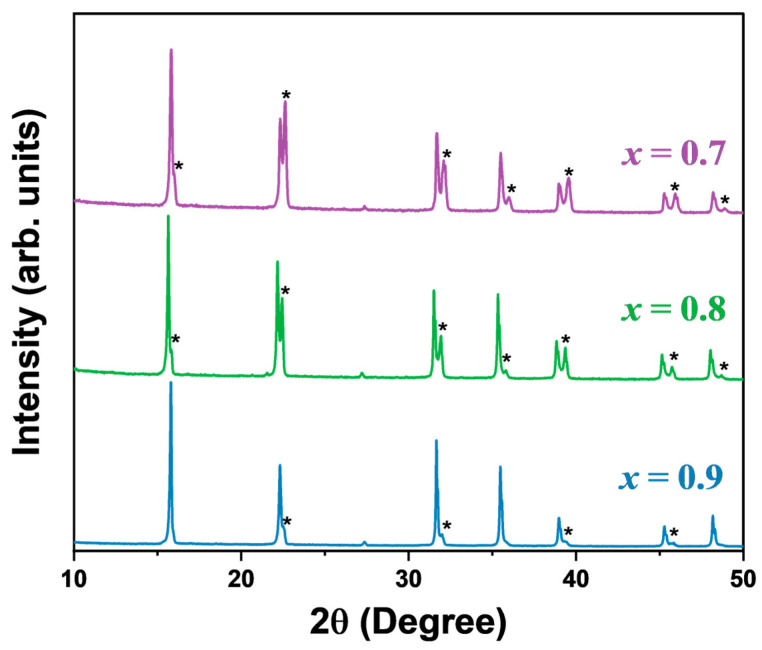
Powder X-ray diffraction patterns of the MA_x_Cs_1−x_PbCl_3_ single crystals (color online). The asterisk denotes the diffraction peaks corresponding to the orthorhombic phase.

Figure 1 presents the powder XRD patterns of the MA_x_Cs_1−x_PbCl_3_ (x = 0.9, 0.8, 0.7) single crystals grown at room temperature. The diffraction peak analysis provides evidence that the high-purity single crystals were successfully synthesized, as no impurity peaks were observed. The sharp and well-defined diffraction peaks indicate that the sample had high crystallinity. At room temperature, CsPbCl_3_ exists in the orthorhombic phase, while MAPbCl_3_ is cubic. However, the XRD patterns of the three mixed crystals demonstrate the coexistence of both the cubic and orthorhombic phases. The asterisks in Figure 1 indicate the diffraction peaks corresponding to the orthorhombic phase. A similar phase coexistence was observed in Cs_x_MA_1−x_PbCl_3_, where the mixed system was synthesized mechanochemically using a hand-grinding method [37]. The cubic phase exhibited relatively pronounced peaks, especially at the highest MA composition (x = 0.9), indicating that the cubic phase was dominant in the mixed crystal. It is not yet known whether phase coexistence is inevitable in the growth of MA_x_Cs_1−x_PbCl_3_ single crystals. The average size of the different phases was calculated from the XRD patterns using a Scherrer analysis in order to gain insights into the phase segregation in our lead halide perovskites. The following Scherrer equation was applied to determine the average size (D):

(1) $$D=\frac{K\lambda }{\beta cos\theta }$$

In this equation, K represents the shape factor (approximately 0.94), λ is the wavelength of the X-ray source (1.5406 Å), β is the full width at the half maximum (FWHM) of the diffraction peak in radians, and θ is the Bragg angle. The calculated sizes are in the order of hundreds of nanometers in both phases, but the average size of the cubic phase is larger than that of the orthorhombic phase. It indicates a dominant cubic phase in the mixed single crystals over the investigated concentration range. The obtained results are presented in Table 1.

The position dependence of the Raman and Brillouin spectra was investigated by using the microscope integrated into each spectrometer. The measured spectra exhibited no discernible positional dependence, as illustrated in Figures S1–S3 in the Supplementary Materials. The probed length scale, namely the beam waist of the laser light at the focal point for the backscattering geometry, was a few µm. This indicates that the length scale of the phase inhomogeneity was smaller than a few µm. This is consistent with the Scherrer analysis results, as suggested in Table 1.

The XRD patterns of MA_x_Cs_1−x_PbCl_3_ with x = 0.9, 0.8, and 0.7 were used to determine the lattice constants, assuming the average symmetry as the dominant cubic phase. The findings revealed that the lattice constants were 5.65 Å, 5.67 Å, and 5.64 Å, respectively. The lattice constants for MA_x_Cs_1−x_PbCl_3_ (x = 1, 0.9, 0.8, 0.7) are shown in Figure S4 alongside the theoretical lattice constant for x = 0 from the DFT calculation [38]. The smaller inorganic Cs cation (1.81 Å) compared to the larger organic MA cation (2.70 Å) [39] may have induced distortions in the cuboctahedral cavity, resulting in a general shrinkage of the cell parameters and a subsequent decrease in the lattice constant with increasing Cs concentration. However, the lattice constant of MA_x_Cs_1−x_PbCl_3_ with x = 0.8 deviated from this decreasing behavior, the origin of which is not yet clear.

### 3.2. Vibrational Properties Probed Using Raman Spectroscopy

**Figure 2:**
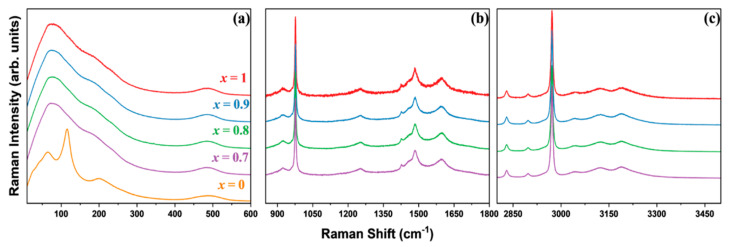
Room temperature Raman spectra of MA_x_Cs_1−x_PbCl_3_ (x = 1, 0.9, 0.8, 0.7, 0) single crystals in three distinct wavenumber ranges: (**a**) 10–600 cm^−1^, (**b**) 850–1800 cm^−1^, and (**c**) 2800–3500 cm^−1^ (color online).

Figure 2 shows the Raman spectra of the MA_x_Cs_1−x_PbCl_3_ single crystals at room temperature, with x values ranging from 0 to 1. The spectrum was recorded over a broad frequency range spanning from 10 to 3500 cm^−1^ and is divided into low, intermediate, and high wavenumber regions. The vibrational modes observed in MA_x_Cs_1−x_PbCl_3_ (x = 1, 0.9, 0.8, 0.7) extend up to 3500 cm^−1^. The high-frequency modes are primarily attributed to the internal vibrations of the MA units. In contrast, high-frequency vibrational modes are absent in CsPbCl_3_ due to the presence of heavy Pb^2+^ and Cs^+^ ions, together with the absence of any internal vibrational modes associated with the inorganic Cs^+^ cation. Figure S5 in the Supplementary Materials depicts the room temperature Raman spectra of the MACl and PbCl_2_ powders to identify the peaks from the MA and lead chloride system. The spectra of these powders indicate different bonding characteristics compared to those of the synthesized mixed single crystals. In the single crystals, the interactions between MA and the lead octahedra resulted in a shift of several peak positions, reflecting the strong interactions and bonding in the three-dimensional lattice structure.

The peak positions of all observed vibrational modes were determined using a curve-fitting analysis with the Lorentzian function. The resulting individual fitting lines for all the synthesized compositions are depicted in Figures S6–S10 in the Supplementary Materials. The mode wavenumbers obtained from the fitting analyses are listed in Table 2, along with their respective mode assignments.

The single crystals of MA_x_Cs_1−x_PbCl_3_ (x = 1, 0.9, 0.8, 0.7, 0) exhibited a crystalline structure that encloses A-site cations within the interstitial spaces of the PbCl_6_ octahedra. Raman spectroscopy revealed different vibrational modes corresponding to the PbCl_6_ octahedra, A-site cation motions, and internal modes of the A-site cation, each covering different frequency ranges. In cubic symmetry, the rotation of the hydrogen-bonded MA^+^ cation within the lead octahedral cage leads to a dynamic disordered arrangement within the lattice. This dynamic disorder can produce structural fluctuations as a consequence of the interaction of the organic cation within the octahedral cages and the PbCl_6_ lattice, thereby activating Raman modes in the average cubic symmetry [47,48]. The low-frequency lattice modes, with a frequency below 600 cm^−1^, included the vibrational modes of the Pb-Cl octahedra and translational motions of the crystal lattice. The torsional mode (τ) of the MA cation was observed at approximately 484~485 cm^−1^. The low-frequency modes exhibited minimal variation across all mixed compositions. However, the lattice mode associated with the rotation of the MA cation observed at 239 cm^−1^ exhibited a decrease in intensity as the cesium concentration increased. This suggests that the incorporation of cesium disrupted the octahedral cavity, thereby influencing the interaction between the organic MA cation and the lead octahedra, consequently affecting its rotational behavior.

The vibrational modes for CsPbCl_3_ were observed at 23, 41, 64, 109, 116, 203, and 488 cm^−1^, which is consistent with previous research [40,43,45]. The modes below 200 cm^−1^ are attributed to the PbCl_6_ octahedra and motions involving the Cs^+^ cation and PbCl_6_ octahedra. In particular, the modes observed at 203 cm^−1^ and 488 cm^−1^ correspond to the first-order and the second-order vibrational modes associated with the translational motion of Cs^+^ cations, as previously reported [40]. In contrast to the organic MA and FA cations, the Cs^+^ cations lack rotational degrees of freedom and internal vibrations, exhibiting head-to-head motion accompanied by Cl face expansion, which contributes to the dynamic disorder observed in these materials [49,50].

In the mid-frequency range, the Raman modes observed at 922 cm^−1^ and 1253 cm^−1^ were associated with the rocking mode of the MA cation, while the mode at 977 cm^−1^ was related to the stretching of the C–N bond. These modes showed no significant variation upon the inclusion of the Cs cation. CH_3_ symmetric and asymmetric bending modes were observed in the range of 1420~1460 cm^−1^, while NH_3_ bending modes were observed between 1480 cm^−1^ and 1600 cm^−1^. At higher frequencies, C–H stretching modes were observed in the range of 2800 cm^−1^ to 3000 cm^−1^, while N–H stretching modes appeared at even higher frequencies. The internal modes of the organic MA cation observed in the medium and high-frequency ranges did not exhibit any discernible changes with varying Cs concentrations. However, the low-frequency lattice modes exhibited near identical characteristics for CsPbCl_3_ and MAPbCl_3_ and appeared to be superimposed or merged for MA_x_Cs_1−x_PbCl_3_ (x = 0.9, 0.8, 0.7) single crystals. Consequently, the inclusion of the Cs cation in the system did not significantly alter the low-frequency lattice modes at room temperature.

The arrangement of A-site cations and their interaction with the crystal lattice significantly influences the macroscopic properties of perovskites. Therefore, it is crucial to investigate the timescale of cation rotations, especially since the vibrational modes of the mixed single crystals did not notably change with the incorporation of the Cs cation. The correlation time, denoted as $\tau_{C}$, represents the duration required for a cation to complete one respective motion of a specific mode. The correlation times were determined using the following equation [50]:

(2) $$\tau_{C}=\frac{1}{2\pi c\omega_{F}}$$

Here, $\omega_{F}$ represents the full width at the half maximum (FWHM) of the relevant Raman mode, and *c* is the speed of light. The value of $\tau_{C}$ was calculated for the low-frequency mode observed at around 122 cm^−1^, the torsional mode, and the C–N stretching modes of the single crystals of MA_x_Cs_1−x_PbCl_3_ (x = 1, 0.9, 0.8, 0.7). The calculated $\tau_{C}$ values for the torsional modes were found to be similar, with values of 72 fs for x = 1, and 71 fs, 72 fs, and 71 fs for x = 0.9, 0.8, and 0.7, respectively. However, the $\tau_{C}$ values for the C–N stretching mode exhibited a notable decrease with increasing cesium, with values of 1001 fs, 913 fs, 927 fs, and 920 fs for x = 1, 0.9, 0.8, and 0.7, respectively. For the low-frequency octahedral mode near 122 cm^−1^, the calculated $\tau_{C}$ was 72 fs, 68 fs, 66 fs, and 68 fs for x = 1, 0.9, 0.8, and 0.7, respectively. All $\tau_{C}$ results are summarized in Table 3. The smaller $\tau_{C}$ values for both the C–N stretching mode and the low-frequency octahedral mode in the mixed crystals compared to pure MAPbCl_3_ suggest that an increased cesium concentration leads to a more compact octahedral space [34]. This structural modification, together with the enhanced disorder due to Cs substitution, affects the rotational dynamics of the MA cation within the lead octahedral frame and its interaction with the lead octahedra.

The analysis of the Raman spectra as a function of the Cs concentration suggests that incorporating Cs causes lattice distortions, as reflected in the changes in the calculated $\tau_{C}$ values. These distortions can be attributed to several factors, including the size mismatch between the inorganic Cs cation and the organic MA cation, as well as their distinct interactions with the PbCl_6_ octahedra. Previous research has demonstrated that the inclusion of cesium alters the bond lengths of Pb-X within the PbX_6_ octahedra [51,52]. The random substitution of the Cs cation into the MA site generates local heterogeneous stresses throughout the lattice, which, in turn, impacts the lattice dynamics and interactions with neighboring PbCl_6_ octahedra. These interactions can modify the Pb-Cl bond length.

### 3.3. Elastic Properties Probed Using Brillouin Spectroscopy

**Figure 3:**
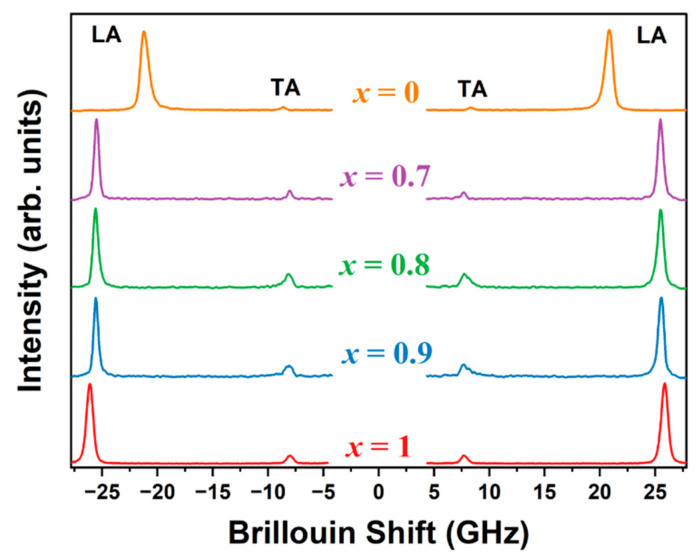
Room temperature Brillouin spectra of MA_x_Cs_1−x_PbCl_3_ (x = 1, 0.9, 0.8, 0.7, 0) single crystals (color online). The LA and TA refer to the longitudinal and transverse acoustic modes, respectively.

**Figure 4:**
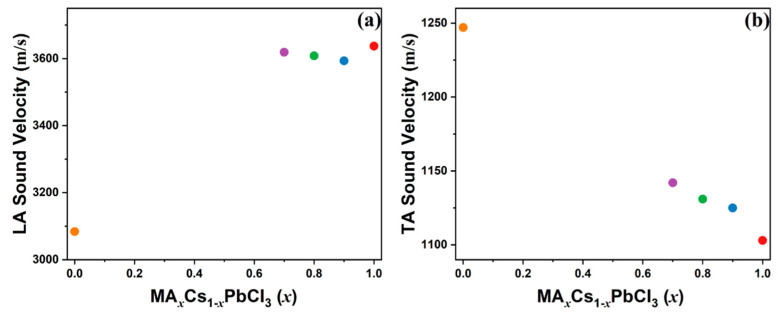
(**a**) Longitudinal acoustic (LA) and (**b**) transverse acoustic (TA) sound velocities as a function of composition x in MA_x_Cs_1−x_PbCl_3_ (x = 1, 0.9, 0.8, 0.7, 0) single crystals (color online).

Figure 3 presents the Brillouin spectra for the mixed single crystals of MA_x_Cs_1−x_PbCl_3_ (x = 1, 0.9, 0.8, 0.7) at room temperature, whereas the spectrum for x = 0 was obtained at 323 K in the cubic phase. All the elastic properties calculated for MA_x_Cs_1−x_PbCl_3_ (x = 0) were derived from the cubic phase observed at 323 K. The spectra display two distinct doublets corresponding to the longitudinal acoustic (LA) and transverse acoustic (TA) modes. The LA mode frequencies shifted to lower values with increasing Cs content, indicating changes in the elastic properties of the perovskite lattice due to Cs cation incorporation. The Brillouin spectra were analyzed using the Voigt function, which is a convolution of the Gaussian instrumental function and the Lorentzian phonon response function. The sound velocity (*V*) was calculated from the acoustic mode frequency ($v_{B}$) using the following relation:

(3) $$V=\frac{\lambda v_{B}}{2n}$$

where *λ* represents the excitation wavelength (532 nm), and *n* represents the refractive index of the crystal. However, the experimental refractive indices for MA_x_Cs_1−x_PbCl_3_ (x = 0.9, 0.8, 0.7) have not been reported. Therefore, we derived approximate refractive indices via linear interpolation between the experimental values of MAPbCl_3_ (*n* = 1.90) single crystals [53] and CsPbCl_3_ (*n* = 1.81) thin films [54], as shown in Figure S11 in the Supplementary Materials. Using the refractive index data, we determined the sound velocities of the LA and TA modes, which are shown in Figure 4. The sound velocity of the LA mode exhibited a slight decrease followed by a gradual increase as the Cs content increased to x = 0.7. However, all values of MA_x_Cs_1−x_PbCl_3_ were significantly higher than the value for x = 0. The longitudinal sound velocity decreased from 3637 m/s to 3084 m/s, approximately a 15% reduction, as the composition changed from x = 1 to x = 0. One possible reason for this difference could be the measurement temperature for CsPbCl_3_, which was higher than RT since its phase is not cubic at RT. The sound velocity of the TA mode exhibited a monotonic increase with increasing Cs content across the investigated composition range. The transverse sound velocity increased from 1103 m/s to 1247 m/s, representing an approximate 12% increase as the composition changed from x = 1 to x = 0.

In this scattering geometry, the LA and TA modes correspond to the elastic constants C_11_ and C_44_, respectively. The elastic constants can be calculated using the sound velocity (*V*) and the crystal density (*ρ*) in the following formula:

(4) $$C_{ij}=\rho V^{2}$$

**Figure 5:**
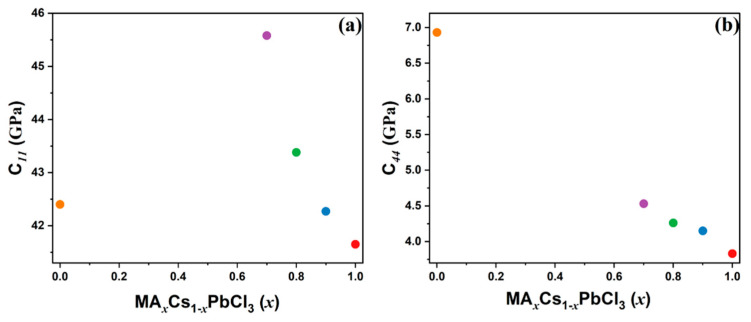
(**a**) C_11_ and (**b**) C_44_ elastic constants as a function of composition in MA_x_Cs_1−x_PbCl_3_ (x = 1, 0.9, 0.8, 0.7, 0) single crystals (color online).

The densities of the MA_x_Cs_1−x_PbCl_3_ (x = 1, 0.9, 0.8, 0.7, 0) single crystals were calculated to be 3149, 3275, 3332, 3479, and 4458 kg/m^3^, respectively, based on the lattice parameters obtained from the PXRD patterns and the chemical formulas. Figure 5a,b shows the elastic constants C_11_ and C_44_ as a function of the MA content. As x varied from 1.0 to 0.7, C_11_ increased from 41.65 GPa to 45.58 GPa, while C_44_ increased from 3.83 GPa to 4.53 GPa. The aforementioned results are summarized in Table 4.

The incorporation of smaller Cs cations into the A site of the perovskite structure resulted in a distortion of the lattice, which in turn produced a nonlinear change in the C_11_ elastic constant. The C_11_ elastic constant demonstrated a monotonic increase until x = 0.7, after which it exhibited a sudden decrease with increasing cesium concentration. In contrast, the C_44_ elastic constant demonstrated a monotonic increase. There could be two competing factors that may have affected the elastic property of the mixed crystal. First, the smaller Cs could have resulted in a more compact and rigid structure, which tends to increase the elastic moduli. Second, the local heterogeneity caused by the random Cs substitution and the consequent perturbation of the N-H bonds of the MA cations in the lattice could have affected the rotational motions of the MA cations, changing their characteristic time scales and increasing the coupling between the acoustic waves and the rotational degrees of freedom. This type of coupling generally reduces the elastic constants. The observed increase in C_11_ and C_44_ with increasing Cs content from 0 to 30% (from x = 1.0 to x = 0.7) suggests that the former effect was dominant. The elastic constants of pure CsPbCl_3_ cannot be compared to those of the mixed crystals because they have been measured at the high temperature of 323 K. This variation in elastic constants is consistent with previous findings on the transition temperature of Cs_x_MA_1−x_PbCl_3_ [37], which decrease with higher Cs content. This indicates that a more rigid Cs_x_MA_1−x_PbCl_3_ structure tends to transform at lower temperatures.

The change in the two elastic constants as a function of varying Cs-MA ratio reveals the complex interplay of the Cs-induced lattice distortions in elastic response to the applied stress. The hydrogen bonding between the MA cation and PbCl_6_ octahedra, which is primarily facilitated through N^+^–H∙∙∙Cl type bonding interactions, may have been affected by the inclusion of smaller Cs cations, resulting in structural distortion. A similar phenomenon was observed in the case of the MA_x_Cs_1−x_PbBr_3_ mixed system, resulting in structural distortions that were responsible for octahedral tilting in this mixed system [52]. Such distortions can alter the material’s stiffness and resistance to deformation, as evidenced by the changes in the elastic constants. This suggests that the smaller size of the Cs cation may have disrupted the crystal structure and modified the hydrogen bonding between the MA cations and halide ions as the Cs concentration increased, resulting in alterations to the Pb-Cl bond lengths and strengths within the inorganic framework of halide perovskites.

## 4. Conclusions

The structural, vibrational, and elastic properties of MA_x_Cs_1−x_PbCl_3_ (x = 1, 0.9, 0.8, 0.7, 0) single crystals were investigated using powder X-ray diffraction and Raman and Brillouin light scattering techniques. The XRD spectra confirmed the successful synthesis of single crystals, indicating the coexistence of both cubic and orthorhombic phases, similar to the previous results shown in Ref. [37]. The length scale of the inhomogeneity caused by phase coexistence was estimated to be less than a few micrometers, as confirmed using micro-Raman and micro-Brillouin techniques. Raman spectroscopy revealed different vibrational modes associated with PbCl_6_ octahedra and A-site cation movements, shedding light on the lattice dynamics influenced by cesium incorporation. It is noteworthy that the incorporation of Cs cations induced lattice distortions, as evidenced by the calculated correlation times and alterations in the Brillouin spectra, leading to changes in the elastic constants and sound velocities. This further suggests that the increase in Cs content resulted in the disruption of the hydrogen bonding between the MA cations and the PbCl_6_ octahedra. The observed increase in both elastic constants as the MA content decreased indicates that the stiffer lattice structure resulting from smaller Cs substitution dominated the elastic properties. All these results highlight the complex relationship between cation size, crystal structure, and lattice dynamics. Further research is required to investigate the relationship between cation dynamics, crystal structure, and optoelectronic performance in order to advance the development of efficient and stable perovskite-based devices.

## Figures and Tables

**Table 1:** Summary of the average sizes (D) of the two different phases in the grown MA_x_Cs_1−x_PbCl_3_ (x = 0.9, 0.8, 0.7) single crystals.

Concentration	Cubic Phase	Orthorhombic Phase
x = 0.9	132 nm	116 nm
x = 0.8	124 nm	105 nm
x = 0.7	100 nm	91 nm

**Table 2:** Mode wavenumbers of all Raman peaks of MA_x_Cs_1−x_PbCl_3_ (x = 1, 0.9, 0.8, 0.7, 0) single crystals measured at room temperature and their mode assignments based on previous studies.

x = 1 (cm^−1^)	x = 0.9 (cm^−1^)	x = 0.8 (cm^−1^)	x = 0.7 (cm^−1^)	x = 0 (cm^−1^)	Mode Assignment
				23	PbCl_6_ vibrations [40]
36	36	36	36	41	PbCl_6_ vibrations [40,41]
63	62	62	63	64	PbCl_6_ vibrations [40,41]
90	89	89	90		*δ_s_* (Cl-Pb-Cl) [42]
				109	PbCl_6_ vibrations [40,43]
				116	PbCl_6_ vibrations [40,43]
122	122	122	123		*δ_as_* (Cl-Pb-Cl) [42]
182	182	182	182		*ν_as_* (Pb–Cl) [42]
				203	First-order phonon mode [40]
239	235	235	234		R^+^ of the MA cation [41]
484	485	485	485		*τ* (MA) [41,44]
				488	Second-order phonon mode [40,45]
922	921	921	921		*ρ* (MA) [41,44]
977	977	977	977		*ν* (C–N) [44]
1253	1253	1252	1253		*ρ* (MA) [46]
1427	1427	1427	1427		*δ_s_* (CH_3_) [44]
1455	1455	1455	1456		*δ_as_* (CH_3_) [44]
1485	1485	1485	1485		*δ_s_* (NH_3_) [46]
1598	1597	1598	1597		*δ_as_* (NH_3_) [46]
2830	2830	2830	2830		*ν_as_* (C–H) [47]
2897	2897	2897	2897		*ν_s_* (C–H) [47]
2947	2946	2947	2947		Symmetric CH_3_ stretch [46]
2972	2972	2972	2972		*ν_s_* (C–H) [47]
3042	3043	3043	3042		*ν_as_* (N–H) [47]
3120	3120	3120	3120		*ν_s_* (N–H) [47]
3189	3189	3190	3190		*ν_s_* (N–H) [47]

*δ*: bending; *ρ*: rocking; *ν*: stretching; *s/as*: symmetric/asymmetric; *τ*: torsion; *R*: rotation.

**Table 3:** Summary of the calculated correlation times $\tau_{C}$ for the three Raman modes of the MA_x_Cs_1−x_PbCl_3_ (x = 1, 0.9, 0.8, 0.7) single crystals.

Raman Mode	x = 1	x = 0.9	x = 0.8	x = 0.7
PbCl_6_ octahedral mode (~122 cm^−1^)	72 fs	68 fs	66 fs	68 fs
Torsional mode (~484 cm^−1^)	72 fs	71 fs	72 fs	71 fs
C–N stretching mode (~977 cm^−1^)	1001 fs	913 fs	927 fs	920 fs

**Table 4:** Summary of the calculated sound velocities and elastic constants for MA_x_Cs_1−x_PbCl_3_ (x = 1, 0.9, 0.8, 0.7, 0) single crystals.

Composition	*V_LA_* (m/s)	*V_TA_* (m/s)	*C*_11_ (GPa)	*C*_44_ (GPa)
x = 1	3637	1103	41.65	3.83
x = 0.9	3593	1125	42.27	4.15
x = 0.8	3608	1131	43.38	4.26
x = 0.7	3619	1142	45.58	4.31
x = 0 ^(a)^	3084	1247	42.40	6.93

^(a)^ measured at 323 K in the cubic phase.

## Data Availability

Data are contained within the article and Supplementary Materials.

## Supplementary Materials

The supporting information can be downloaded at: https://www.mdpi.com/article/10.3390/ma17122862/s1.
