# Supplementary material for: The Effect of Cesium Incorporation on the Vibrational and Elastic Properties of Methylammonium Lead Chloride Perovskite Single Crystals
Source: Materials (Basel). 2024 Jun 12;17(12):2862. doi: 10.3390/ma17122862 (PMC11204745; doi:10.3390/ma17122862)
Supplement: Supplementary file 1 [file materials-17-02862-s001.zip › materials-3014505-supplementary.pdf]

Supplementary Information

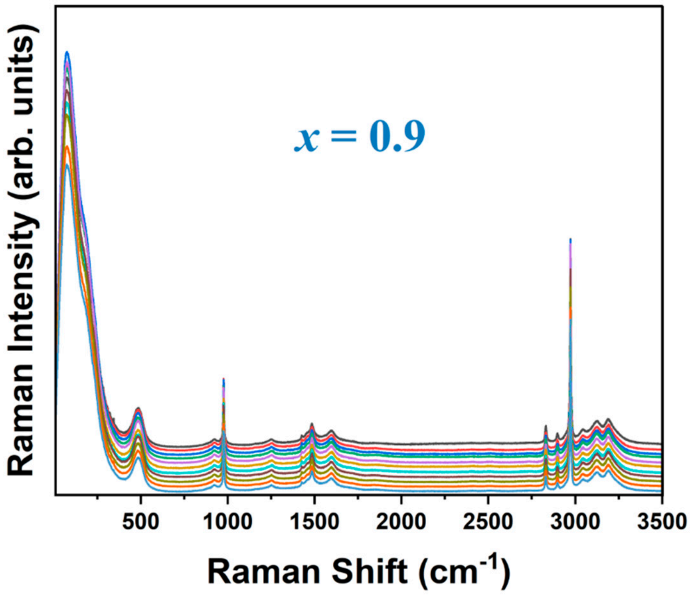

Figure S1. Positional dependence Raman spectrum measurements of $MA_{0.9}Cs_{0.1}PbCl_3$ single crystals at room temperature (color online).

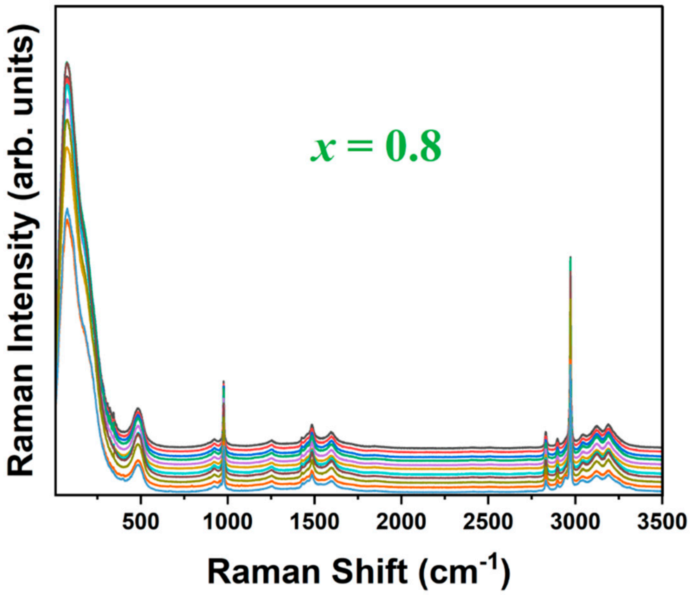

Figure S2. Positional dependence Raman spectrum measurements of $MA_{0.8}Cs_{0.2}PbCl_3$ single crystals at room temperature (color online).

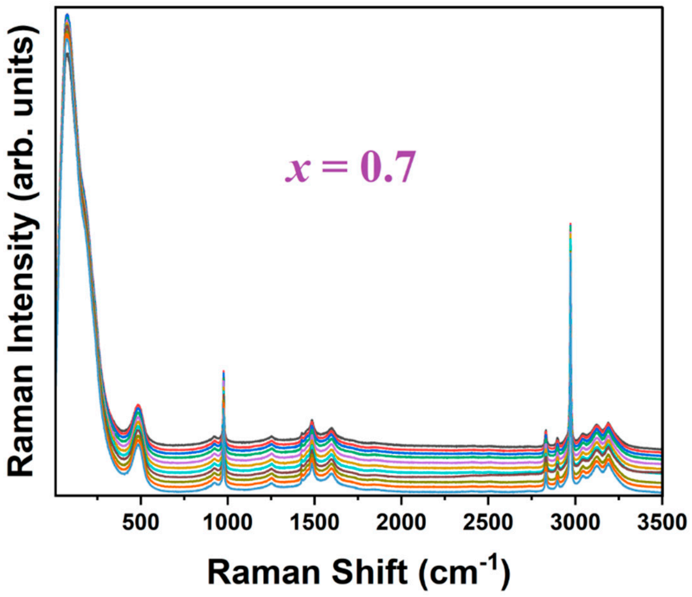

Figure S3. Positional dependence Raman spectrum measurements of $MA_{0.7}Cs_{0.3}PbCl_3$ single crystals at room temperature (color online).

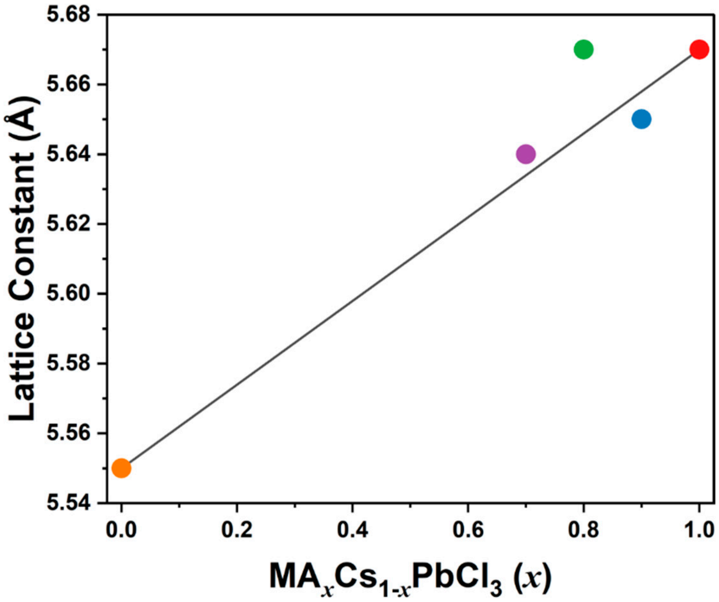

Figure S4. Lattice constant as a function of concentration of the $MA_xCs_{1-x}PbCl_3$ (x = 1, 0.9, 0.8, 0.7) mixed system. The lattice constant for $CsPbCl_3$ was taken from Ref. [38].

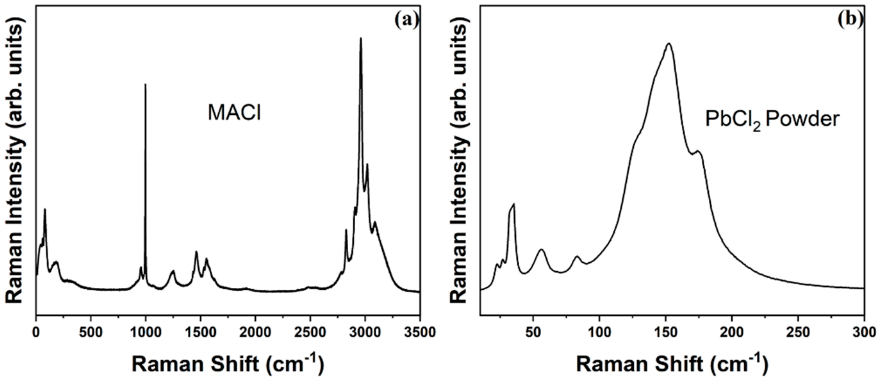

Figure S5. Raman spectra of the (a) MACl and (b) $PbCl_2$ powders at room temperature (color online).

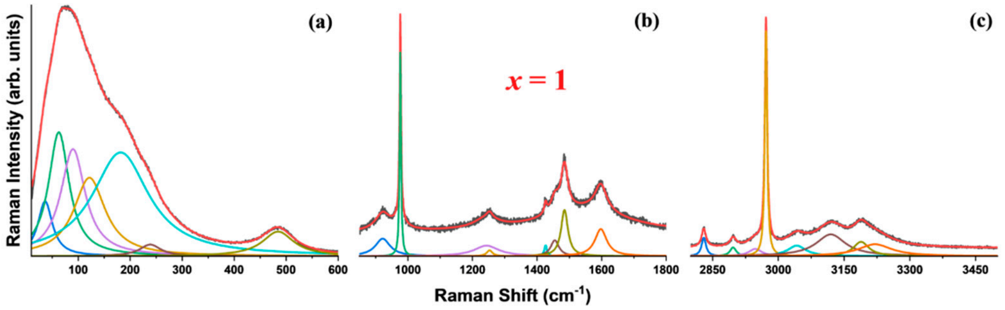

Figure S6. (a–c) Raman spectra and best-fitted curves of $MA_{0.9}Cs_{0.1}PbCl_3$ single crystals at room temperature (color online).

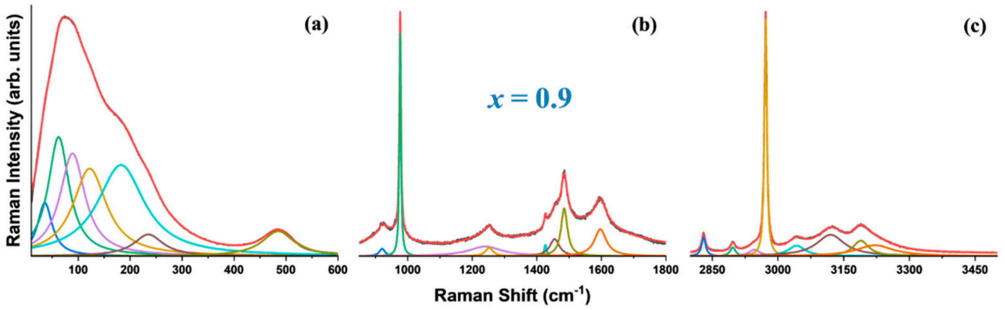

Figure S7. (a–c) Raman spectra and best-fitted curves of $MA_{0.9}Cs_{0.1}PbCl_3$ single crystals at room temperature (color online).

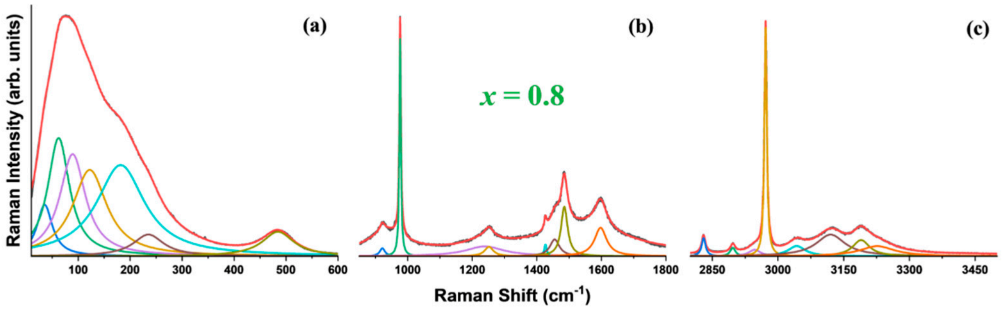

Figure S8. (a–c) Raman spectra and best-fitted curves of $MA_{0.8}Cs_{0.2}PbCl_3$ single crystals at room temperature (color online).

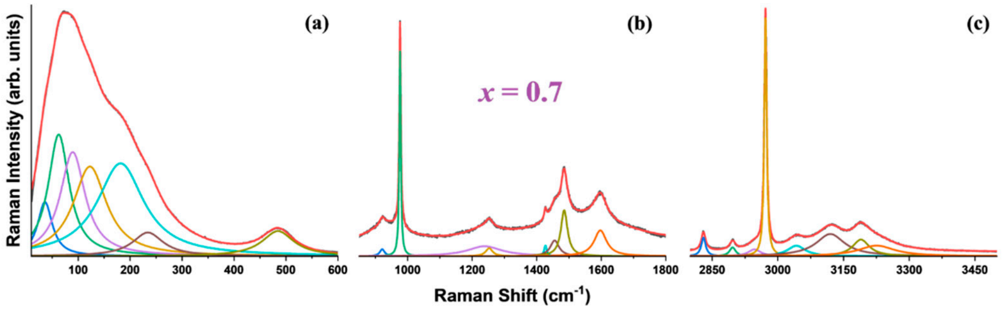

Figure S9. (a–c) Raman spectra and best-fitted curves of $MA_{0.7}Cs_{0.3}PbCl_3$ single crystals at room temperature (color online).

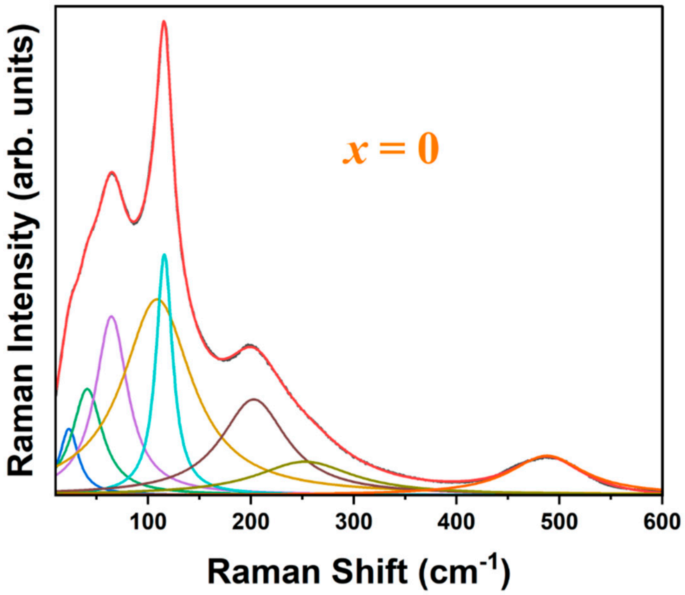

Figure S10. Raman spectra and best-fitted curves of $CsPbCl_3$ single crystals at room temperature (color online).

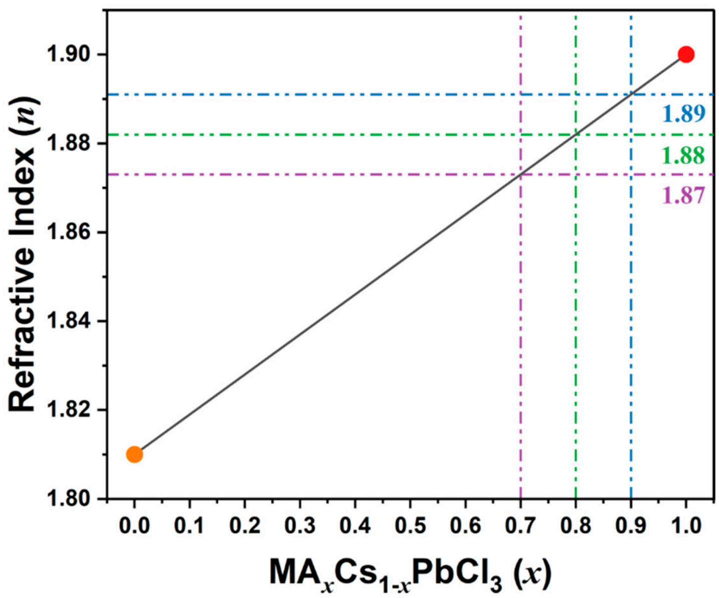

Figure S11. Interpolated refractive index for $MA_xCs_{1-x}PbCl_3$ single crystals. The value for the refractive index for $MAPbCl_3$ [53] and $CsPbCl_3$ [54] were interpolated to obtain values for all mixed compositions (color online).
